# Understanding the flexion-relaxation phenomenon in non-specific chronic low back pain patients throught immersive virtual reality feedback approach

**DOI:** 10.1038/s41598-024-65983-5

**Published:** 2024-07-10

**Authors:** Kevin Rose-Dulcina, Margaux Dubessy, Stéphane Armand, Stéphane Genevay

**Affiliations:** 1 Laboratory of Kinesiology, Geneva University Hospitals and University of Geneva, Geneva, Switzerland; 2 Campus Biotech, Geneva, Switzerland; 3 Division of Rheumatology, Department of Medecine, Geneva University Hospitals, Geneva, Switzerland

**Keywords:** Predictive markers, Bone quality and biomechanics, Musculoskeletal system

## Abstract

The flexion-relaxation phenomenon (FRP) is frequently absent among non-specific chronic low back pain (NSCLBP) patients. However, it is unknown whether this absence is intrinsic to their pathology or merely a consequence of reduced trunk flexion. Immersive virtual reality (IVR) can create a patient avatar whose range of motion can be modulated to differ from the real movement. The present study enrolled 15 NSCLBP patients and 15 asymptomatic participants with similar characteristics to disentangle the relationship between range of motion and the FRP in NSCLBP using IVR. Trunk kinematics and lumbar muscle electromyography were assessed. The IVR environment was combined with a motion capture system to create avatars that moved like each participant. The IVR display showed a closed room and a mirror reflecting the subject’s avatar with a target line to be reached by trunk flexion. The avatar’s trunk movements were modulated from reality, leading the participants to flex their trunk more than their voluntary maximum trunk flexion. Under IVR conditions, NSCLBP patients significantly increased their trunk flexion angle, which was coupled with a significant improvement in the FRP. The absence of the FRP among the NSCLBP population appeared to be primarily related to reduced trunk flexion.

## Introduction

Non-specific chronic low back pain (NSCLBP) is a complex disorder where pain and disability are influenced by many factors, including from the social, psychological and biophysical dimensions^1^. This multiplicity of factors makes the NSCLBP population very heterogeneous. Despite this, one biophysical finding is consistent across all studies on the topic: the disruption of the flexion-relaxation phenomenon (FRP) that is usually observed during trunk forward bending^2^. The FRP is defined as the reduced activity of the lumbar extensor muscles in standing maximum trunk flexion^2^. Often quantified using a flexion-relaxation ratio (FRR), the phenomenon is frequently absent among NSCLBP patients^3^. In other words, NSCLBP patients present greater muscle activity than asymptomatic individuals at maximum trunk flexion. This muscle activity has been hypothesised as a neuromuscular phenomenon limiting range of motion (ROM) and increasing the load on the spine among these patients^4^. As such, the FRP has been proposed as an interesting biomarker for NSCLBP^5^. Some have suggested that the FRP could be the consequence of the stimulation of stretch receptors in posterior discoligamentous tissues during the flexed posture, receptors that act to reflexively inhibit the erector spine muscles^2^. Other authors then suggested that the absence of the FRP could be the consequence of the absence of stimulation for these receptors because of the reduced lumbar ROM observed in NSCLBP patients attempting to protect their back from pain during forward flexion^2,6,7^. Indeed, we previously reported a significant correlation between reduced maximum trunk ROM and the absence of the FRP in an NSCLBP population^6^.

Psychological factors have also been shown to play an important role in the persistence of NSCLBP^8^. Pain induces negative emotions and feelings, which can, in turn, influence pain perception, beliefs about pain’s consequences and behaviours for coping with it^9^. One of the main psychological factors related to NSCLBP is the fear of movement, known as kinesiophobia^9^. Kinesiophobia has previously been reported as being associated with altered lumbar movements^10,11^ and muscle activity^12,13^ in NSCLBP patients, especially during trunk forward bending^14,15^. Perception also seems to influence spine movement, with previous reporting that spinal movement can be modulated by feedback from immersive virtual reality (IVR), both among asymptomatic participants (APs)^16,17^ and among the NSCLBP population^18,19^. Hence, IVR offers the possibility of disconnecting a subject’s perceived ROM from their real ROM, causing a kinaesthetic drift thanks to the modified visual feedback they receive in the direction of the movement suggested to them visually^16^. Only a small kinaesthetic drift can be induced among asymptomatic populations before the subject perceives it, probably due to the already high ROM^18^. The range of manipulation might be more important among NSCLBP patients because of their lower ROM, which is caused by variable muscle guarding, potentially amplified by kinesiophobia^18^.

The present study’s primary objective was to investigate maximum trunk ROM’s influence on the FRP among NSCLBP patients and APs using IVR manipulations. As a prerequisite, we examined previous results from studies in the literature on trunk ROM and the FRP, and we then tested several of their hypotheses to achieve our study’s objective.

Firstly, we expected NSCLBP patients only to exhibit a greater maximum trunk ROM when the IVR feedback showed less movement than the patient’s movement under real conditions (hypothesis 1). Secondly, if hypothesis 1 was validated, we expected only the NSCLBP patients to present with an improved FRP (as evidenced by a lower FRR), correlating with an improved ROM (hypothesis 2). Thirdly, we expected NSCLBP patients to show a significant correlation between greater ROM under IVR conditions and their level of kinesiophobia (hypothesis 3).”

## Method

### Ethics approval and consent to participate

This prospective cohort study was approved by the Research Ethic Cantonal Commission of the Geneva University Hospitals (reference CER: 2020-02152) and conducted in accordance with the principles outlined in the Declaration of Helsinki. Written informed consent was obtained from all participants prior to their inclusion in the study.

### Participants

Fifteen NSCLBP patients and 15 APs were enrolled and then evaluated in a human movement laboratory. Patients were recruited in the rheumatology department of the Geneva Universtity Hospitals in Switzerland. They were included in the NSCLBP group if they had presented with NSCLBP for more than three months (with an absence of infection, rheumatological or neurological diseases, spinal fractures, any known spinal deformities, tumours or radicular symptoms). APs were included in the AP group if they had no history of back pain in the last six months. For both groups, additional exclusion criteria were pregnancy, age below 18 or above 60 years old, previous back surgery, a body mass index over 30, and pain or injury in any other body parts.

### Instruments

The FRP was assessed using active surface electromyography electrodes (Trigno AVANTI, Delsys Inc., Boston, MA, USA) sampling at 1000 Hz. These were positioned bilaterally on the erector spinae longissimus of each participant at the L1 level of the spinous process on abraded skin cleaned with alcohol^20^.

**Figure 1:**
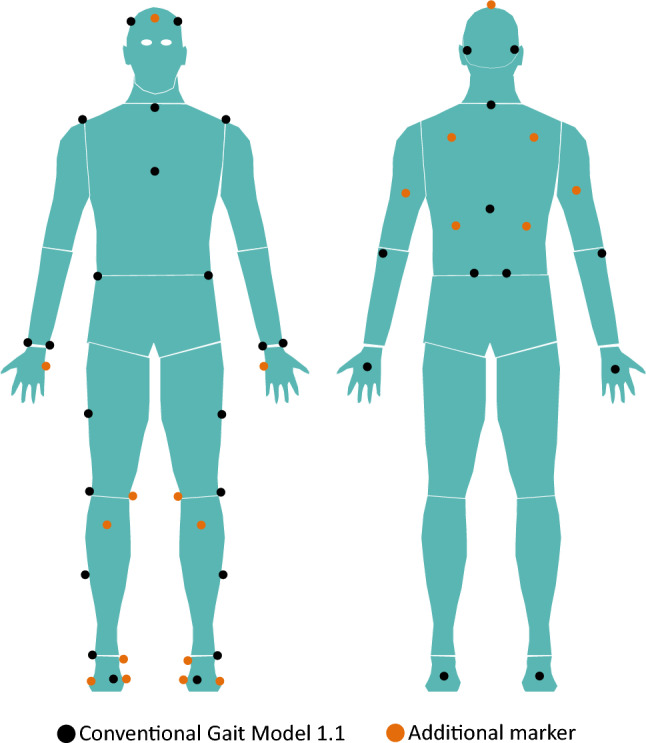
Marker set used for motion capture and virtual reality.

Kinematics were assessed using a 12-camera motion analysis system (Qualisys Oqus7 + , Gothenburg, Sweden) sampling at 100 Hz. Each participant was equipped with 34 reflective markers (14 mm diameter spheres), per the conventional gait model (1.1)^21^. To set up the skeleton for live-streaming and to integrate the avatar’s body into the VR application, 22 additional markers were placed on the participant according to the skeleton set-up documentation for the skeleton motion-capture system (see Fig. 1).

**Figure 2:**
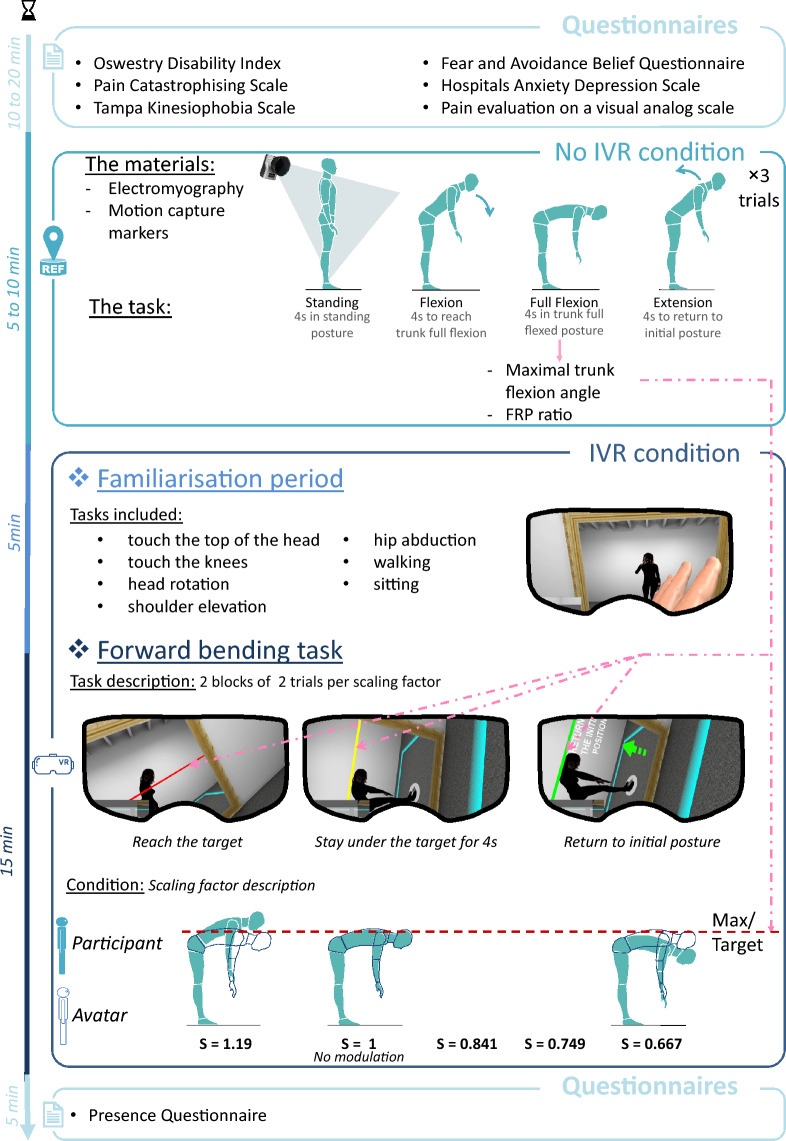
General diagrammatic overview of the study methodology. *IVR* immersive virtual reality, *FRP* flexion-relaxation phenomenon, *S* scaling factor.

The IVR system was composed of an IVR headset (HTC Vive, HTC Corporation, Taoyuan, Taiwan) and an IVR application (developed in Unity software, version 2019.2.7, Unity Technologies, San Francisco, Carlifornia, USA). This application displayed an IVR environment composed of a neutral room, an avatar of the participant and a ‘mirror’ on one side to reflect the avatar. The mirror included a red target line, adjusted for each participant to reach (see Fig. 2). Real-life data of the participant’s skeleton movements, taken from the movements of the 56 markers by the motion capture system, were live-streamed to the IVR application. The movements of the avatar thus matched those of the participant, who received visual feedback through the IVR headset. Because of the first-person visual feedback, the avatar embodied the participant (see Fig. 2).

### Procedure

On arrival, to better characterise our population, all the NSCLBP patients completed the oswestry disability index (ODI)^22^, the pain catastrophizing scale (PCS)^23^, the tampa scale of kinesiophobia (TSK)^24^, the fear-avoidance belief questionnaire (FABQ)^25^, and the hospital anxiety and depression scale (HADS)^26^. Due to the nature of these questionnaires, APs only completed the HADS. All participants were equipped with reflective markers and electromyography electrodes. The trial itself involved patients bending forward, as far as possible, with their legs straight. This movement was composed of four phases, with each phase lasting 4 s: standing (static), flexion, full flexion (static), and return to the standing position. Thus, the participant began in a static standing position for 4 s, then took 4 s to bend forward to reach their maximum flexion keeping their legs straight (flexion), they remained static at their maximum flexion for 4 s (full flexion), and, lastly, took 4 s to return to their initial standing position (Fig. 2). Three consecutive trials were performed, and an audible metronome was used to regulate the timing of their movements. The maximum trunk flexion angle was extracted from these trials and used to create the personalised target in the IVR environment.

Participants were then equipped with the IVR headset and were given a familiarisation period of five minutes and a set of standardised movements (touch the top of the head, touch the knees, head rotation, shoulder elevation, hip abduction, walking, sitting) to help them explore their virtual interaction with their avatar (Fig. 2). Next, the participants performed the identical maximum trunk forward bending trials with their legs straight, but in the IVR environment and under different IVR feedback conditions. IVR feedback conditions modulated the avatar’s trunk movement in the headset, increasing or decreasing the visual perception of the participant’s real-life movements. Participants were not made aware that this modulation process was possible; they were simply instructed to bend until the top of their trunk (the C7 marker) moved below the red target line (Fig. 2). As soon as the avatar’s trunk passed under the target line, it turned from red to yellow, and participants were instructed to stay flexed for four seconds until the line turned to green, signalling that they had succeeded the test and could slowly return to their initial position (Fig. 2). If so, the trial will be considered as a “successful trial”.

After their IVR tests, each participant completed the Presence Questionnaire^27^ to quantify how involved and immersed they had felt in the VR environment. Finally, the participants were informed about their avatar’s movement modulations and were questioned about their perceptions of any movement discrepancies.

### Modulation of the avatar’s movement

IVR feedback conditions were defined by a scaling factor used to modulate the avatar’s live-streamed trunk movements. Scaling factors were chosen based on previous work by Roosink et al.^18^ on NSCLBP patients in the military. Because that study had aimed to increase its participants’ ROM, most of the scaling factors selected reduced the avatar’s trunk movement compared to the participants’ real movement. Scaling factors ≥ 1 were also selected so that participants did not only react to scaling factors reducing their avatar’s trunk movements, which could have made them aware of the modulation. The scaling factors are presented in Table 1. Each participant performed two blocks of two trials per scaling factor in an order randomly assigned using MATLAB R2021a software’s ‘randperm’ function (The MathWorks, Inc., Natick, MA, USA).

**Table 1:** The scaling factors selected and the number of trials per block.

Avatar movement characteristics	Scaling factor	Number of trials, block 1	Number of trials, block 2	Number of movements
Less flexion than reality	0.667	2	2	4
	0.749	2	2	4
	0.841	2	2	4
Equal to reality	1	2	2	4
More flexion than reality	1.190	2	2	4
Total		10	10	20

The motion capture skeleton was used to determine the local rotation and position of every segment, starting from the root segment (the pelvis). The marker-based skeleton of the participant created by the motion capture system was applied to two avatars in the Unity software: the embodiment avatar and the target avatar (the mirror reflection of the embodiment avatar). The embodiment avatar followed the participant’s true movements to avoid motion sickness. The scaling factor was only applied to the target avatar.

As the subject begins to bend, their embodiment avatar follows their movements exactly (without the scaling factor), while the target avatar follows their movements with the scaling factor chosen for the trial. The rotation of the embodiment avatar and target avatar were updated in every new frame. The delay between the subject’s movement in the real world and the movements of the embodiment avatar and the target avatar in the virtual world depends on the communication between the QTM (Qualisys Track Manager 2018.1 build 4180, Gothenburg, Sweden) and Unity software packages. As this study used a dedicated network and the Unity application’s frame-refresh rate was 90 Hz, the total delay experienced by the subject ranged between 20 and 100 ms. For a rotation between 0° and 20°, the target avatar root segment (the spine) rotation was multiplied by the scaling factor. Beyond 20°, the target avatar’s shoulders also rotated according to the difference between the embodiment avatar’s and the target avatar’s spine rotation in order to keep the correct visual feedback (shoulders were oriented according to the target avatar’s back movements).

This operation modified the real-time motion capture rotation, resulting in a lesser rotation in the target avatar when the scaling factor (s) was less than 1. The reduced rotation followed the same direction as the current motion capture rotation. When the scaling factor was equal to 1, no scaling was applied, and the operation simply returned the real-life motion capture rotation.

### Data processing

As per Gutierrez et al.^28^, the thorax segment was defined using the C7, T10, xiphoid process and jugular notch markers. The *x-*axis (*x*) represents the posterior–anterior direction and was defined as the normalised vector from the midpoint between the xiphoid process and T10 to the midpoint between the jugular notch and C7. The *y-*axis represents the left–right direction and was orthogonal (to the left) to the plane formed by these four markers, with the *x-*axis being the result of the cross-product of *y* and *z*. Thorax flexion, therefore, refers to movement along the y-axis (left–right); thorax obliquity refers to movement along the x-axis (posterior–anterior); and thorax rotation indicates axial rotation around the z-axis with respect to the frame of the laboratory (a standard right-handed Cartesian coordinate system with a fixed-point origin) during the trunk forward bending task.

The raw electromyography signals recorded during the procedure were filtered using a Butterworth (4th order) pass-band filter (20–500 Hz) and were then full-wave rectified and low-pass filtered (2.5 Hz) to produce linear envelopes^29^. Gouteron et al.^30^ suggested using the FRR proposed by Xia et al.^31^. However, this FRR is very sensitive to any potential low values (a relaxed muscle) in the denominator. The present study used an FRR presenting AUC, sensitivity, specificity and Youden Indexsimilar to the suggested one. The FRR was calculated for the erector spinae longissimus, on both sides, as follows^6,29,32^:

1 $$FRR=\frac{1s {RMS}_{fullflexion}}{1s {RMS}_{flexion}}$$,

where RMS is the maximum root mean square of the linear envelope of one second during the flexion and full flexion phases. A lower FRR indicated a greater state of muscle relaxation.

### Outcome parameters

The primary outcome was the maximum trunk sagittal flexion angle. This parameter was calculated under No IVR and IVR conditions. Under No IVR conditions, the maximum angle achieved during the three trials was used in the analysis and as the target to reach under IVR conditions (Fig. 2). The maximum angle achieved during the IVR trials was used in the analysis under IVR conditions.

Secondary outcomes were the FRR, the TSK score, the trunk angle gain and the percentage of successful IVR trials. FRR was calculated using the same trials retained for calculating the angles mentioned previously. Because the FRR had been reported to be asymmetric between the left and right sides^6,32^, the higher FRR was used for statistical analysis^32,33^. Trunk angle gain refers to the difference between the maximum angle under No IVR conditions and under IVR conditions. The percentage of successful IVR trials (target reached) in each group of participants was compared with reference to the scaling factors.

### Statistical analysis

The Shapiro–Wilk test was used to confirm the data distribution’s normality, and then individual characteristics were compared between groups using unpaired Student’s t-tests and Pearson’s chi-squared tests for dichotomous outcomes.

Due to the non-normal distribution of the trunk angles, the FRR group comparisons were performed using the Wilcoxon rank sum test (for unpaired data), and condition comparisons were performed using the Wilcoxon signed-rank test (for paired data). Results are reported as median [interquartile range].

Spearman coefficient correlation analysis was used to quantify the association between trunk ROM gain and the intensity of kinesiophobia as measured using the TSK. For all tests, a p-value < 0.05 was considered significant.

## Results

### General characteristics and questionnaires

No significant differences were observed between the two groups’ general characteristics, except that NSCLBP patients had a significantly higher anxiety score (Table 2). Mean Presence Questionnaire scores in the IVR environment were between 70 and 80 (satisfactory^34^) for both groups, with no significant differences between them. None of the participants reported having noticed a difference between their own movements and the target avatar’s movements observed in the mirror under any of the IVR conditions.

**Table 2:** General characteristics of the study sample.

	NSCLBP patients (n = 15)	Asymptomatic participants (n = 15)	*p*-value	95% confidence interval	Effect size
**Participant characteristics**					
Female (n)^k^	7 (47)	7 (47)	1.00	− 0.3 to 0.3	0.043
Age (years)^t^	39.9 ± 8.6	39.7 ± 11.1	0.954	− 7.8 to 8.3	0.024
Height (cm)^t^	172.7 ± 7.5	173.9 ± 6.9	0.678	− 7.0 to 4.6	0.172
Weight (kg)^t^	68.9 ± 14.9	70.4 ± 12.6	0.78	− 12.7 to 9.6	0.116
BMI (kg.m^2^)^t^	23.0 ± 4.0	23.2 ± 3.2	0.882	− 3.1 to 2.75	0.061
**Pain-related characteristics**					
Current pain (VAS/10)^t^	38.3 ± 27.6	–	–	–	–
Pain duration (years)^t^	5.9 ± 3.9	–	–	–	–
Oswestry Disability Index (%)^t^	14.2 ± 7.7	–	–	–	–
Tampa Scale of Kinesiophobia^t^	16.2 ± 10.3	–	–	–	–
Pain Catastrophizing Scale^t^	25.6 ± 12.0	–	–	–	–
HADS anxiety^t^	9.4 ± 4.7	5.38 ± 3.1	0.017*	0.8 to 7.35	1.058
HADS depression^t^	5.1 ± 3.9	2.62 ± 3.1	0.082	− 0.3 to 5.4	0.744
Presence Questionnaire^t^	71.2 ± 12.5	74.77 ± 13.1	0.488	− 13.9 to 6.8	0.287

NSCLBP, non-specific chronic low back pain; VAS, visual analog scale; HADS, hospital anxiety and depression scale; ^k^, values are n (%) and Pearson Khi^2^ test was performed; ^t^, values are mean ± standard deviation and/or unpaired Student’s t-test was performed; *p < 0.05.

### Prerequisites

**Figure 3:**
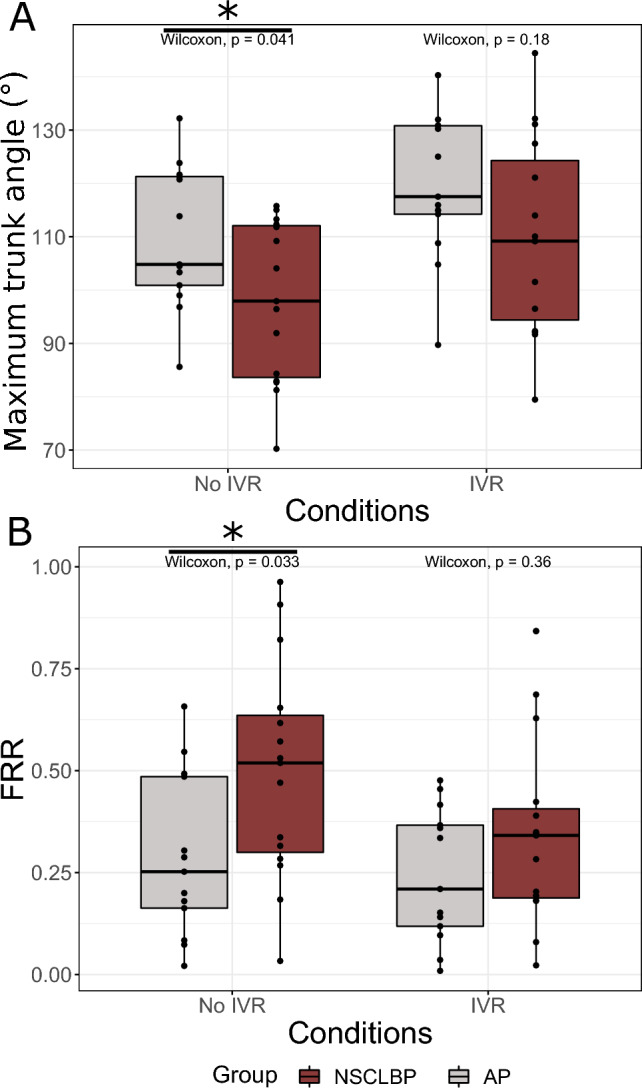
Group comparisons of trunk (**A**) kinematic and (**B**) electromyography parameters. FRR, flexion-relaxation ratiol Wilcoxon, Wilcoxon rank sum testl NSCLBP, non-specific chronic low back pain; AP, asymptomatic participants; IVR, immersive virtual reality.

Concerning group comparisons under No IVR conditions, NSCLBP patients had a significantly lower voluntary maximum trunk flexion angle (NSCLBP = 97.9° [83.6–112.1]; APs = 104.8° [100.9–121.3]; *p* = 0.041) and a significantly greater FRR compared to APs (NSCLBP = 0.53 [0.31–0.64]; APs = 0.25 [0.16–0.48]; *p* = 0.033) (Figs. 3A and B, respectively).

### Group comparisons

NSCLBP patients had significantly more successes (reaching the red target line) in the IVR trials than the APS (NSCLBP = 93.8 ± 1.3%; AP = 86.3 ± 2.0%) The results under each IVR condition are represented in Supplementary Material 1.

Under the reduced feedback condition, no significant differences were found between the groups for either maximum trunk flexion angle (NSCLBP = 109.2° [94.4–124.3]; APs = 117.5 [114.2–130.9]; *p* = 0.180) or the FRR (NSCLBP = 0.34 [0.20–0.41]; APs = 0.21 [0.12–0.37]; *p* = 0.360) (Figs. 3A and B, respectively).

### Condition comparisons

**Figure 4:**
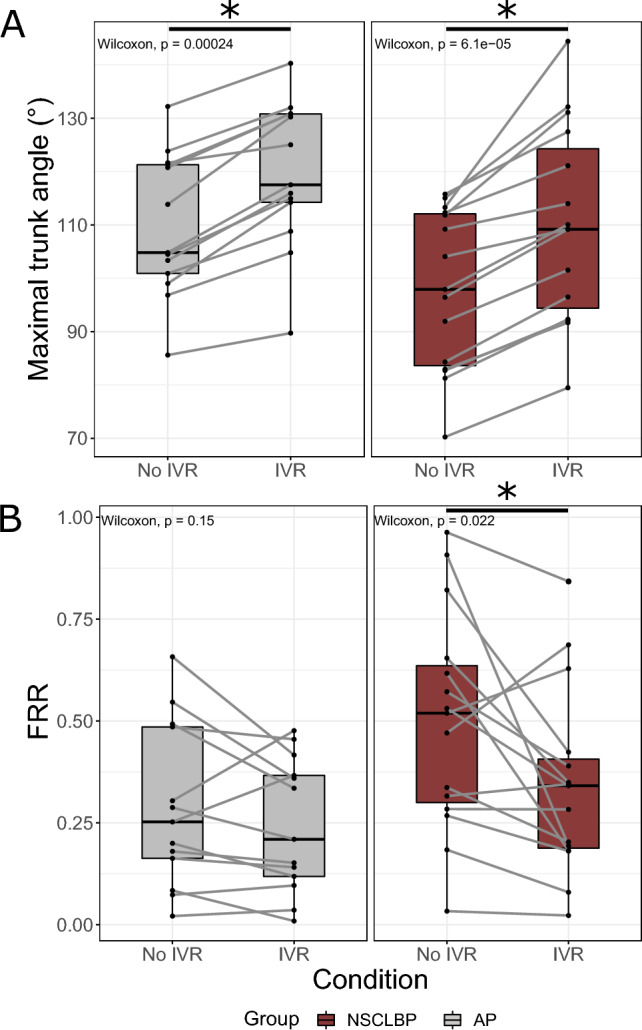
Condition comparisons for trunk (**A**) kinematic and (**B**) electromyography parameters. FRR, flexion-relaxation ratio; Wilcoxon, Wilcoxon signed-rank test; NSCLBP, non-specific chronic low back pain; AP, asymptomatic participants; IVR, immersive virtual reality.

Regarding comparisons between the No IVR and IVR conditions, an increase in maximum trunk flexion angle was observed under the IVR condition among NSCLBP patients (No IVR = 97.9° [83.6–112.1]; IVR = 109.2° [94.4–124.3]; *p* < 0.001) and APs (No IVR = 104.8° [100.9–121.3]; IVR = 117.5° [114.2–130.9]; *p* < 0.001) (Fig. 4A, hypothesis 1 confirmed).

A significant decrease in the FRR was only observed among NSCLBP patients (No IVR = 0.53 [0.31–0.64]; IVR = 0.34 [0.20–0.41]; *p* = 0.022) (Fig. 4B, hypothesis 2 confirmed). Data on the different parameters per group and condition are provided in Supplementary Material 2.

### Correlation between gain in ROM and kinesiophobia

No significant correlation was found between gains in the ROM and the TSK among NSCLBP patients (r = − 0.17; *p* = 0.548) (Supplementary Material 3, hypothesis 3 unconfirmed).

## Discussion

The present study’s main objective was to investigate the relationship between ROM and the FRP in NSCLBP patients. Our results confirmed those in the literature. Under the No IVR condition, the NSCLBP patients included in this study had a smaller maximum trunk flexion angle and a lower FRP (i.e. a greater FRR) than the APs^3,6^. By unconsciously dissociating the perceived movement of their avatars from their real-life movements during the IVR experiment, the NSCLBP group was able to significantly increase their maximum trunk flexion angles (hypothesis 1). By doing so, we observed a significant decrease in the FRR, corresponding to an FRP improvement in this population (hypothesis 2). Moreover, under IVR conditions, no significant differences were observed between the groups for these two outcomes. However, no significant correlation was found between the gain in real-life ROM and the level of kinesiophobia (hypothesis 3).

### Trunk flexion angle

Under IVR conditions, NSCLBP patients showed an increase in voluntary maximum trunk flexion angle over the No IVR condition (hypothesis 1). However, this was also observed among the APs, which was not expected. This improvement in ROM suggests that a subject will not always push to reach their mechanical limits when they are simply asked to perform a maximum trunk forward bend. One explanation, for both groups, could be the numerous repetitions performed under the IVR condition, whereas ROM tests without IVR were always recorded at the beginning of each experiment. The repetition of flexion movements may have acutely stretched passive structures and led to an increase in ROM^35^. Additional psychological factors may also have contributed to a greater ROM, as visual feedback increases motivation and could lead to better performance^36–38^.

The presence of kinesiophobia in the NSCLBP group could be related to this result. This has been proposed as an important factor in modulating trunk ROM among NSCLBP patients, particularly during trunk forward flexion^15^. One explanation for the increase in ROM is that the altered visual feedback from the avatar might limit the effects of kinesiophobia by biasing the subject’s visual self-perception and leading not only to an increase in ROM but also to a greater possibility of increasing it. This could also explain the higher percentage of successful trials among NSCLBP patients than among APs, and this could mean that APs have increased their ROM under IVR conditions but with more difficulty. However, the present study could not confirm this interaction as no statistically significant correlation could be found between a gain in ROM and the TSK score among NSCLBP patients (hypothesis 3). Several reasons might explain this finding. Among them, our study was performed on a small group of individuals and was not powered to detect such an effect. Moreover, our NSCLBP group presented lower kinesiophobia scores than participants in a previous study^15^. Also, the TSK might not be specific to trunk flexion kinesiophobia^39^.

### Flexion-relaxation phenomenon

As expected, our NSCLBP patients presented with a lower FRR and an increased ROM under IVR conditions (hypothesis 2). These results were consistent with the hypothesis formulated by Colloca et al.^2^, which associated the FRP with stimulation of the spine’s stretch receptors in the flexed posture, “acting to reflexively inhibit the erector spine muscles”. Under the No IVR condition, the angle of trunk flexion among NSCLPB patients was insufficient to stimulate the stretch receptors and provoke the FRP. However, under IVR conditions, NSCLBP patients were able to increase their voluntary maximum trunk flexion angle, leading to such a decrease in FRR that variations between the two groups were no longer statistically different. A study using VR-modulated visual feedback reported similar results for patients with neck pain^40^. The stretch receptors in our NSCLBP patients were perhaps stimulated enough to trigger the relaxation of their spine muscles (as shown by their lower FRR). This, too, is in line with previous results reporting an improvement in the FRP after therapies such as flexibility exercises^41,42^. Unfortunately, our results suggest discarding the use of the FRP as a biomarker of NSCLBP, as suggested elsewhere^5^, as they did not support the absence of the FRP as being a marker of an intrinsic muscular dysfunction. Rather, the results suggested a secondary phenomenon simply reflecting the partially self-limited ROM in these patients.

The unexpected increase in trunk ROM among APs did not lead to a decrease in their FRR, which was already low under the No IVR condition. Indeed, as the FRP had already been triggered by the ROM initiated under No IVR conditions, the additional gain in ROM under IVR conditions may not have been able to significantly modify this phenomenon.

### Limitations

The present study had some limitations. First, the tasks performed between the conditions with and without IVR were not identical. Indeed, during forward bending under IVR conditions, patients had to look at their avatar as if they were looking in a mirror to their left, implying a rotation of the head that might influence paraspinal muscle activation. Secondly, no specific timings were imposed under IVR conditions, whereas four-second phases were imposed using a metronome under No IVR conditions. This may also have influenced the results for the FRR^43^. Finally, the ROM under No IVR conditions was conducted without wearing the head-mounted display, and we cannot ascertain whether the additional load (550 grammes) may have modified paraspinal muscle activation. However, to the best of our knowledge, these elements have only been reported to influence the timings of muscle activation/deactivation^44^. No effects on ROM or the FRP have been reported. It is of note that the NSCLBP patients included in this study presented with low levels of kinesiophobia as measured using the TSK. It is possible that repeating the experiment among a larger group of patients with a higher level of kinesiophobia would demonstrate an interaction between kinesiophobia and the ROM achievable under IVR conditions. Further experiments will be necessary to better understand kinesiophobia’s effects on ROM in this population, perhaps also using IVR.

## Conclusion

Using visual feedback from an immersive virtual reality (IVR) system enabled us to dissociate perceived and actual trunk flexion range of motion (ROM) and significantly increase the ROM in a population with non-specific chronic low back pain (NSCLBP). This also resulted in an improvement in the flexion-relaxation phenomenon (FRP). This strongly suggests that the absence of the FRP in this population is caused by a trunk flexion ROM so small that it does not allow the activation of the stretch receptors. The present study does not support the use of the FRP as a biomarker of NSCLBP. However, by permitting an unconscious dissociation between real ROM and perceived ROM, IVR appears to be a promising tool with which to explore and better understand neuromuscular modifications among NSCLBP patients.

## Supplementary Information

Supplementary Information 1.

Supplementary Information 2.

Supplementary Information 3.

## Data Availability

The datasets analysed during the current study are available from the corresponding author upon reasonable request.
