# Author Correction: Understanding the flexion-relaxation phenomenon in non-specific chronic low back pain patients through immersive virtual reality feedback approach

**DOI:** 10.1038/s41598-024-71957-4

**Published:** 2024-09-09

**Authors:** Kevin Rose-Dulcina, Margaux Dubessy, Stéphane Armand, Stéphane Genevay

**Affiliations:** 1 Laboratory of Kinesiology, Geneva University Hospitals and University of Geneva, Geneva, Switzerland; 2 Campus Biotech, Geneva, Switzerland; 3 Division of Rheumatology, Department of Medecine, Geneva University Hospitals, Geneva, Switzerland

Correction to: *Scientific Reports* 10.1038/s41598-024-65983-5, published online 10 July 2024

The original version of this Article contained an error in the name of Kevin Rose-Dulcina, Stéphane Armand, and Stéphane Genevay, which were incorrectly given as Rose-Dulcina Kevin, Armand Stéphane, and Genevay Stéphane.

The original Article has been corrected.

