# Supplementary material for: Understanding the flexion-relaxation phenomenon in non-specific chronic low back pain patients throught immersive virtual reality feedback approach
Source: Sci Rep. 2024 Jul 10;14:15936. doi: 10.1038/s41598-024-65983-5 (PMC11236989; doi:10.1038/s41598-024-65983-5)

**Supplementary material 1:** Percentage of successful trials for each immersive virtual reality condition (mean ± standard deviation). NSCLBP is non-specific chronic low back pain; AP is asymptomatic participants


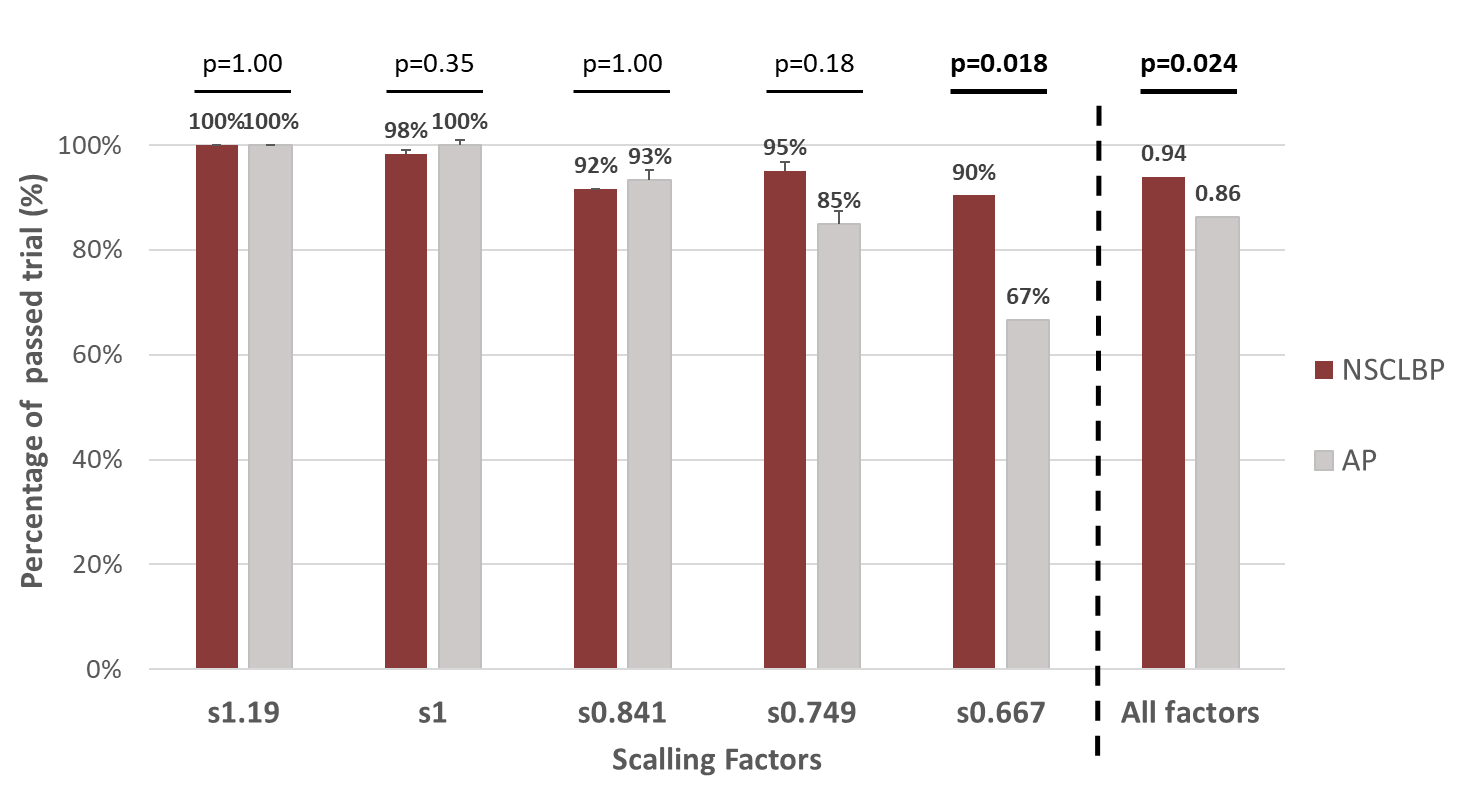

Supplement: Supplementary file 1 — Supplementary Information 1. [file 41598_2024_65983_MOESM1_ESM.docx]
