# Supplementary material for: Understanding the flexion-relaxation phenomenon in non-specific chronic low back pain patients throught immersive virtual reality feedback approach
Source: Sci Rep. 2024 Jul 10;14:15936. doi: 10.1038/s41598-024-65983-5 (PMC11236989; doi:10.1038/s41598-024-65983-5)
Supplement: Supplementary file 2 — Supplementary Information 2. [file 41598_2024_65983_MOESM2_ESM.docx]

**Supplementary material 2**: Intra and inter-group comparisons for kinematic and electromyography parameters

| **Group** | **NSCLBP (n=15)** | **AP (n=15)** | **p-value of Inter-group comparison** |
| --- | --- | --- | --- |
| **Maximal trunk flexion angle (°)** | | | |
| No IVR | 97.9  [83.6 : 112.1] | 104.8  [100.9 : 121.3] | ***0.041*** |
| IVR | 109.2  [94.4 : 124.3] | 117.5  [114.2 : 130.9] | *0.180* |
| **p-value of Inter-condition comparison** | **<0.001** | **<0.001** |  |
| **Trunk angle difference between conditions (°)** | | | |
| Gain | -11.0  [-12.5 : -9.0] | -9.5  [-12.6 : -8.0] | *0.294* |
| **FRR** | | | |
| No VR | 0.53  [0.31 : 0.64] | 0.25  [0.16 : 0.48] | ***0.033*** |
| IVR | 0.34  [0.20 : 0.41] | 0.21  [0.12 : 0.37] | *0.360* |
| **p-value of Inter- condition comparison** | **0.022** | 0.150 |  |

*Results are presented as median (interquartile range); NSCLBP: nonspecific low back pain, AP: asymptomatic participants, FR ratio: flexion-relaxation ratio; IVR: immersive virtual reality.*
