# Supplementary material for: Understanding the flexion-relaxation phenomenon in non-specific chronic low back pain patients throught immersive virtual reality feedback approach
Source: Sci Rep. 2024 Jul 10;14:15936. doi: 10.1038/s41598-024-65983-5 (PMC11236989; doi:10.1038/s41598-024-65983-5)

**Supplementary material 3:** relationship between Tampa Scale of Kinesiophobia score and the gain in ROM in immersive virtual reality conditions. R = spearman coefficient; *p* = p-value


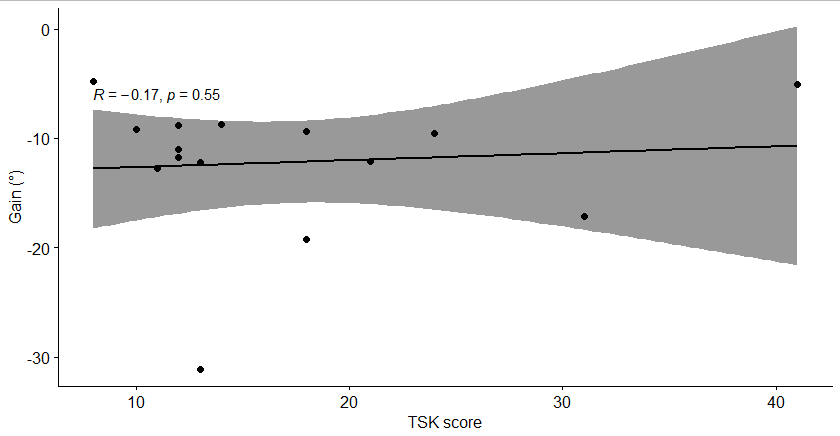

Supplement: Supplementary file 3 — Supplementary Information 3. [file 41598_2024_65983_MOESM3_ESM.docx]
